# Effect of level of infection by gastrointestinal nematodes and anthelmintic treatment on milk yield in dairy sheep

**DOI:** 10.1051/parasite/2021068

**Published:** 2021-10-21

**Authors:** María Martínez-Valladares, Elías Martín-Ramos, Myriam Esteban-Ballesteros, Rafael Balaña-Fouce, Francisco Antonio Rojo-Vázquez

**Affiliations:** 1 Instituto de Ganadería de Montaña (CSIC-Universidad de León) Grulleros 24346 León España; 2 Departamento de Sanidad Animal, Facultad de Veterinaria, Universidad de León Campus de Vegazana 24071 León España; 3 Sociedad Cooperativa Limitada Bajo Duero (COBADU) Moraleja del Vino 49150 Zamora España; 4 Departamento de Ciencias Biomédicas, Facultad de Veterinaria, Universidad de León 24071 León Spain

**Keywords:** Dairy sheep, Gastrointestinal nematode, Anthelmintic treatment, Milk yield

## Abstract

The effects of gastrointestinal nematode infections and anthelmintic treatment on milk yields was compared between flocks with a low level (LL) of eggs per gram (epg) before partum and with a high level (HL). Faecal egg count reduction tests (FECRTs) were carried out before partum comparing a treated group with netobimin with an untreated group. Ewes belonging to LL flocks produced 55.4% more milk than ewes from HL flocks. A negative correlation was found between the mean epg before treatment and the mean milk yield per flock (*r* = −0.860; *p* < 0.01). However, treated ewes produced 10.1% more milk than untreated ewes in LL flocks, although in HL flocks, treated ewes produced less milk (−2.7%). The treatment of flocks even with low levels of infection can improve the milk yields. In this study, the epg before partum had a greater influence on total milk yield than the anthelmintic treatment.

## Abstract (fr)

Les effets sur les rendements laitiers des infections à nématodes gastro-intestinaux et d’un traitement anthelminthique ont été comparés entre des troupeaux avec un bas niveau (BN) d’œufs par gramme (opg) avant la mise bas et d’autres avec un haut niveau (HN). Un test de réduction du nombre d’œufs dans les selles (FECRT) a été effectué avant la mise bas en comparant un groupe traité avec de la nétobimine avec un groupe non traité. Les brebis appartenant aux troupeaux BN ont produit 55,4 % de lait de plus que les brebis des troupeaux HN. Une corrélation négative a été trouvée entre l’opg moyen avant traitement et le rendement laitier moyen par troupeau (*r* = −0,860 ; *p* < 0,01). Cependant, les brebis traitées ont produit 10,1 % de lait de plus que les brebis non traitées dans les troupeaux BN, bien que dans les troupeaux HN les brebis traitées aient produit moins de lait (−2,7 %). Le traitement des troupeaux, même ceux avec de faibles niveaux d’infection, peut améliorer les rendements laitiers. Dans cette étude, l’opg avant mise bas a eu une plus grande influence sur la production totale de lait que le traitement anthelminthique.

## Introduction

Infections by gastrointestinal nematodes (GIN) affect grazing ruminants, and especially small ruminants, around the world [5]. According to Torres-Acosta et al. [17], a balanced grazing system provides not only nutrients but also an average GIN burden, resulting in optimal levels of productivity; however, when this balance is broken, the result is an increase in the worm burden and inevitable production losses. Coop and Kyriazakis [8] proposed that sheep respond to parasitism by relocating feed resources, with higher priority to maintaining vital body function, followed by other functions such as growing, reproduction and lactation and, as a result, dedicating fewer resources to functions related to immune response to control the infection.

Although the negative impact of GIN on production parameters such as milk yield is known, there are not many studies specifically quantifying the consequences of the infection under field conditions. Evaluating the effect of an anthelmintic treatment on lactation in grazing animals can be challenging when this effect is compared between different flocks. In this sense, husbandry practices could also have an impact and therefore could be responsible for the high variability among flocks. Under experimental conditions, Cruz-Rojo et al. [10] showed that the treatment with a controlled-release albendazole bolus of Spanish Assaf ewes infected with the GIN *Teladorsagia circumcincta* after lambing, led to an increase of 11% of total milk yield when compared to the untreated group.

In this context, the aim of the present study was to assess the impact on milk yield of an anthelmintic treatment with netobimin before lambing on eight commercial dairy sheep farms with different levels of infection by GIN.

## Materials and methods

### Selection of flocks and GIN burden

The study was conducted in the northeast of Spain with the collaboration of the livestock cooperative COBADU (Cooperativa Bajo Duero). The flocks did not receive any anthelmintic treatment 3 months prior to their selection and were under a semi-extensive system in which animals graze on pastures for 6–8 h per day and the rest of the time are kept indoors. Therefore, the first step was the identification of those farms infected by GIN by means of a faecal egg count (FEC). Individual faecal samples from the rectum of 10 animals were randomly taken in each flock. Faecal samples were analysed individually by a modified McMaster technique using a saturated solution of sodium chloride (density = 1.2 g/mL) for egg counting. The detection limit of this technique was 15 eggs per gram (epg). In total, 8 flocks naturally infected by GIN were included in the study, 4 of them with a low level of infection (LL; mean FEC < 150 epg) and 4 with a high level of infection (HL; mean FEC > 400 epg). This classification was performed according to the mean FEC of the infected flocks by GIN in the area of study.

The morphological identification of the GIN species infecting the flocks included in the study was performed on third stage larvae after coprocultures were performed.

### Faecal egg count reduction test (FECRT)

Faecal egg count reduction (FECR) tests were carried out in all flocks around 3–4 weeks before partum. To do this, two groups of ewes were composed of 10 animals each. A single dose of netobimin (Hapasil^®^, MSD Animal Health, Spain) was administered orally at the recommended dose (7.5 mg/kg body weight) to the treated group (TG), and the control group (CG) was treated at the same time with a placebo solution. Each animal was weighed before treatment in order to adjust the individual dose. Rectal faecal samples were taken from every ewe on the day of treatment (day 0) and 10–14 days post-treatment. All samples were processed and analysed individually by a modified McMaster technique, as previously described. The reduction in egg number was calculated using the following formula [7]:

$$\mathrm{FECR}\left(\%\right)=100\times \left(1-\left(\mathrm{Mean}\mathrm{FEC}\mathrm{day}10-\frac{14}{\mathrm{Mean}}\mathrm{FEC}\mathrm{day}0\right)\right).$$

Resistance is present when the percentage reduction in FEC is less than 95% and the 95% confidence level is less than 90%. If only one of the two criteria is met, resistance is suspected [7].

### Milk yield

With the aim of providing suckling lambs with colostrum, the assessment of milk production was done monthly starting in the second month of the lactation and ending at day 120 post-lactation. Therefore, a total of four determinations were obtained, at months 2, 3, 4, and 5 of lactation. The monthly production was estimated as the mean of two consecutive determinations, multiplied by two milkings per day and by 30 days between two determinations. The total yield (kg) throughout the experiment was calculated as the sum of all consecutive periods (months 2–3, 3–4, and 4–5).

### Milk composition

The milk content of fat and protein was measured at the same time as the milk yield by the automated infrared method using a Milko Scan 104 (NS N. Foss Electric, Hillerad, Denmark) calibrated against known sample standards.

### Statistical analyses

FEC determination before partum and total milk yield at the end of lactation were compared between flocks using Student’s test. The correlation between these two variables was measured by the Pearson’s correlation coefficient. Moreover, a multivariate linear regression analysis was conducted to assess the association between the milk yield and the percentage of each GIN species before treatment.

A generalized linear model (GLM) repeated measure analysis of the effect of the level of infection (LL and HL flocks) or anthelmintic treatment (CG and TG groups) on milk production throughout the study was performed. In particular, milk production values were introduced into the model as the dependent variable and the level of infection or the treatment as between-subject effects, using four-way repeated-measures.

Values of *p* < 0.05 were considered significant. All tests were carried out using the SPSS software program for Windows.

## Results and discussion

Previous studies have shown that GIN infections can have adverse effects on production, especially in relation to losses in weight gain rates and milk production; that said, one must also consider the large variations in management practices and environment. In this study, we included 8 dairy flocks belonging to the Assaf breed in order to determine the influence of GIN infection during peripartum as well as the administration of an anthelmintic treatment, netobimin, on total milk production at the end of lactation. Initially the flocks included in this study were classified according to the GIN infection level in the last third of gestation, low (FEC: 102–147 epg) or high (FEC: 426–567 epg). The mean FEC on day 0 (treatment day) was 140 ± 31 epg and 465 ± 66 epg in LL and HL, respectively, showing significant differences between them (*p* < 0.0001). However, a FEC threshold for production limiting infections of 500 epg was proposed for cases where *Haemonchus contortus* was absent [6]. The GIN species identified in these flocks were *T. circumcincta* (mean: 60.0%; 19.8–100.0%), *Trichostrongylus* spp (mean: 29.4%; 0.0–58.0%) and *Chabertia ovina* (mean: 10.1%; 0.0–23.8%); the presence of *Haemonchus contortus*, the most pathogenic GIN species, was not detected in any flock.

After netobimin administration, all flocks were found to be susceptible to this drug with a FECR value higher than 95% and 95% confidence level higher than 90%. With the aim of determining the influence of FEC on total milk yield, this parameter was compared between LL and HL flocks but only the group of untreated ewes (CG) was, taken into consideration to omit the drug influence on milk yield. At the end of the study the mean total of milk production per ewe was significantly higher in those belonging to LL flocks (226 ± 71 kg) when compared to HL flocks (101 ± 33 kg) (*p* < 0.0001); consequently, ewes with a low level of infection before partum produced 125 kg more milk than ewes with a high level of infection, representing a 55.4% increase. A significant and negative correlation was found between the mean FEC before treatment and the mean milk yield per flock in untreated animals at the end of lactation (*r* = −0.860; *p* < 0.01). No influence of GIN species composition on milk yield was found. In addition, and according to the GLM analysis, the level of infection had a significant influence on the monthly milk yield throughout the study (*p* = 0.038), confirming the adverse impact of GIN infections on dairy flocks. In a review about the interaction between parasitism and milk production, it was concluded that infected ewes produced an average 22% less of milk than uninfected animals [18]. Suarez et al. [16] suggested that even short periods of exposure to a subclinical GIN infection could have a negative effect on the milk yields in dairy sheep. In other species such as goats and cattle, the same reduction in yields of infected animals was reported [4, 11].

**Figure 1:**
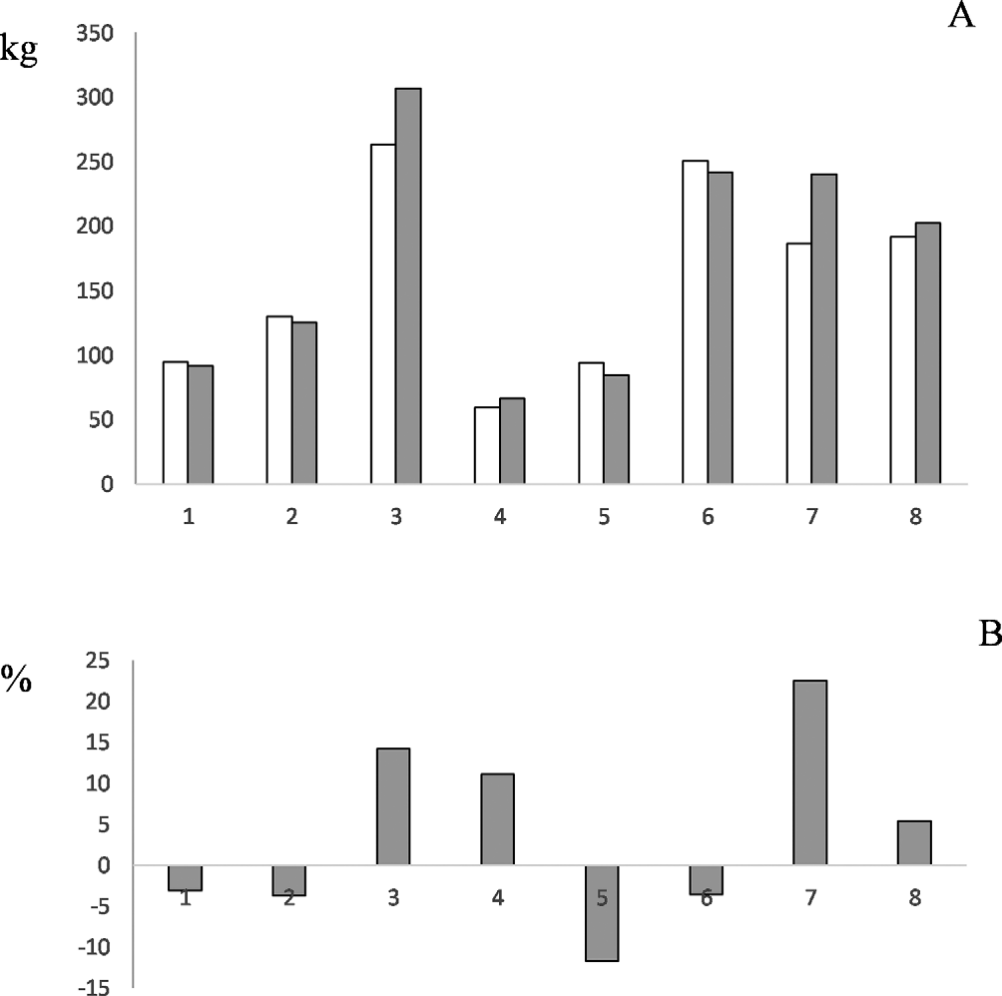
(A) Total milk yield at the end of lactation (kg) per flock and group; control (white) and treated (grey) groups. (B) Increase in milk production in the treatment group with respect to the control group.

In relation to the effect of netobimin treatment on milk yield, this parameter was calculated in the two groups of each flock, TG and CG. As a result, in 4 out of 8 flocks the TG produced more milk at the end of lactation than CG, with a mean increment of 13.3% (ranging between 5.4 and 22.4%) in these flocks. However, in the other flocks (4/8), the TG produced 5.5% less milk than CG (ranging between −3.1 and −11.7%) (Fig. 1). Regardless of the flock, the mean milk yield per ewe at the end of lactation was 170 ± 80 and 180 ± 95 kg in ewes belonging to CG and TG, respectively; therefore, treated ewes produced 5.6% more milk than untreated ewes. However, according to the GLM analysis, the treatment did not have a significant influence on the monthly milk yield, nor were there any differences found between CG and TG in total production at the end of lactation. In a similar study, Fthenakis et al. [12] analysed the milk yield in a group of ewes treated before lambing with a different benzimidazole, albendazole; in that case, the yield at the end of lactation in the treated group was 7.4% more than in the untreated group. Interestingly in that same study, when a second albendazole treatment was administered after lambing, it was found that milk production had increased by 18.5%. Sechi et al. [15] showed that the administration of a single dose of albendazole after lambing led to an increment of 7% in total milk yield in the treated group when compared to a control group. All these studies show the beneficial effect of anthelmintic treatment in naturally infected flocks.

We also determined the consequence of anthelmintic treatment on milk yield by taking into account the level of infection of the flock during peripartum. Surprisingly, in LL flocks, the TG produced 10.1% more milk (248 ± 43 kg) than the CG (223 ± 40 kg). However, in HL flocks, the TG produced (92 ± 25 kg) less milk than the CG (94 ± 29 kg) (−2.7%). Furthermore, it is important to note that the administration of an anthelmintic treatment before lambing in flocks with a low level of GIN infection, can improve total milk yield. The lack of treatment effect in HL flocks could be due to the fact that ewes continued grazing on the same pastures after treatment, resulting in reinfections, high levels of epg, and lower milk production.

In the current study, the percentage of milk fat and milk protein was measured monthly in two LL flocks (flocks 6 and 7) simultaneously with milk production. The mean percentage of milk fat in flock 6 ranged from 4.7% to 7.8% and between 5.4 and 6.7% in flock 7. With regards to the percentage of milk protein, these values were within the range of 4.3–5.2% in flock 6 and 4.4–5.2% in flock 7. No differences were shown between groups in the percentage of fat or protein. Although the number of flocks in which these parameters are measured is very limited, according to our results, netobimin treatment did not affect the percentage of milk fat or milk throughout lactation. These results are consistent with other studies in which the administration of albendazole after lambing did not affect the milk composition during lactation [12]. However, in other studies conducted in goats, protein and fat contents were influenced by breed, level of FEC and milking days [1]. Again, in dairy goats, a significant increase of milk yield (12%), fat (29.9%), protein (23.3%) and lactose (19.6%) was observed in animals treated with an anthelmintic [14].

In the current study, the FEC before partum had a greater influence on total milk yield than the administration of an anthelmintic treatment. It is important to note that the final efficacy of the anthelmintic treatment on milk production is a consequence of several factors, including parasite-related factors such as worm burden and/or GIN species infecting animals, host-related factors such as breed, nutritional status or individual resistance to the parasite, and even external factors such as epidemiology of the GIN species, climatic conditions or season of treatment [3, 9, 13]. In lactating ewes in Spain, most treatments are administered before partum due to the withdrawal periods during lactation. With the aim of improving the control of GIN infection, and only with the absence of anthelmintic resistance, animals could be moved after treatment to “clean paddocks”, meaning with low GIN infectivity, to avoid reinfections and to reduce worm burden during lactation. On the other hand, this cannot be followed when anthelmintic resistance has already been shown in a flock, since the progeny of resistant parasites surviving treatment will contribute disproportionately to the next generation of parasites [2].
